# Genetic flux over time in the *Salmonella *lineage

**DOI:** 10.1186/gb-2007-8-6-r100

**Published:** 2007-06-04

**Authors:** Georgios S Vernikos, Nicholas R Thomson, Julian Parkhill

**Affiliations:** 1 The Wellcome Trust Sanger Institute, Wellcome Trust Genome Campus, Hinxton, Cambridge CB10 1SA, UK

## Abstract

**Background:**

DNA sequences that are shared between closely related organisms while being absent from their common ancestor and from sister lineages of that ancestor are likely to have been acquired by horizontal gene transfer. Over time, the composition of those sequences tends to become more similar to the compositional signature of their host (amelioration).

**Results:**

From a whole-genome comparative analysis of eleven *Salmonella*, three *Escherichia coli *and one *Shigella *strain, we inferred the relative time of insertion of putative horizontally acquired (PHA) genes in three *Salmonella *strains on different branches of the *S. enterica *phylogenetic tree. Compositional analysis suggests that most of the PHA genes are still undergoing an amelioration process and shows a clear correlation between time of insertion and the level of amelioration.

**Conclusion:**

The results show that older insertions include almost all functional classes. However, very recent horizontal transfer events in the *Salmonella *lineage involve primarily prophage elements that are shared only between very recently diverged lineages; despite this, the prophage sequence composition is close to that of the host, indicating that host adaptation, rather than amelioration, is likely to be the source of the compositional similarity. Almost half of the PHA genes were acquired at the base of the *Salmonella *lineage, whereas nearly three-quarters are shared between most *S. enterica *subspecies. The numerical distribution of PHA genes in the *Salmonella *tree topology correlates well with the divergence of the major *Salmonella *species, highlighting the major impact of horizontal transfer on the evolution of the salmonellae.

## Abstract 2

From a whole-genome comparative analysis of *Salmonella*, *Escherichia coli *and *Shigella *strains, the relative time of insertion of putative horizontally acquired genes in three *Salmonella *strains were inferred, highlighting the major impact of horizontal transfer in the evolution of the salmonellae.

## Background

The divergence of *Salmonella *and *Escherichia coli *lineages from their common ancestor has been estimated to have occurred approximately 100-140 million years (Myr) ago [1,2]. Using models of amelioration to estimate the time of horizontal gene transfer (HGT) events it has been previously shown [3] that the entire *E. coli *chromosome contains more than 600 kb of horizontally transferred, protein-coding DNA. The same authors estimated the HGT rate to be 31 kb per million years, which is close to the point mutation frequency. Under this assumption the *E. coli *and *Salmonella enterica *lineages have each gained and lost more than 3 megabases (Mb) of novel DNA since their divergence.

DNA sequences of recent HGT events can deviate strongly from the genome background composition while older insertions have often lost their donor-specific sequence signature [3]. Generally, each genome exhibits a reasonably constant background sequence composition; however, some genes, traditionally considered part of the core-gene dataset, such as rRNA and ribosomal protein-coding genes, often deviate compositionally from the genome background sequence composition mainly due to specific, well-preserved functional constraints rather than their alien origin (although some of them can be horizontally acquired [4,5]). In those cases the effect of the amelioration over time is expected to be trivial since strong selection applies.

Base composition and specifically G+C content is known to be related to phylogeny [6]. Consequently, closely related organisms tend to have similar G+C content; for example, the average G+C content of *E. coli*, *Shigella *and *Salmonella *lineages is approximately 50%, 51% and 52%, respectively, while for the Gram-positive *Staphylococcus *and *Streptococcus *lineages the average G+C content is 33% and 38%, respectively.

Usually horizontally acquired genes are introduced into a single lineage, and, therefore, the acquired DNA sequence will be limited to the descendents of the recipient strain and absent from closely related ones. For example, *Salmonella *Pathogenicity Island (SPI) 1, a 40 kb island carrying a type-III secretion system that enabled the invasion of epithelial cells is present in both *Salmonella *species, *S. bongori *and *S. enterica*, while it is absent from the genome of *E. coli*. Consequently SPI-1 represents an ancient HGT event that took place close to the divergence of the two genera (*E. coli *and *Salmonella*) [7]. On the other hand, SPI-2, which is important for systemic infection, is a mosaic of two independent acquisitions [8]: the tetrathionate reductase (ttr) gene cluster, a 15 kb region (present in *S. bongori *and *S. enterica*); and a 25 kb region encoding an additional type-III secretion system (present only in *S. enterica*). Consequently, using a reference tree topology, HGT events can be distributed into increasing depth phylogenetic branches; moreover, we can infer their relative time of insertion, that is, the most ancient branch in the tree topology that shares a putative horizontally acquired (PHA) gene present only in descendant lineages. Based on this principle, Daubin and Ochman [9] identified sequences unique to monophyletic groups at increasing phylogenetic depths, and studied the characteristics of sequences with no detectable database match (ORFans) using *E. coli *MG1655 as a reference genome.

A key step in inferring the relative time of insertion of PHA genes is the construction of phylogenetic trees that will capture reliably the evolutionary history of the organisms at hand. rRNA genes have been extensively used as molecular chronometers for inferring the phylogeny and building tree topologies [10]. However, it has been shown that even these traditionally core components of the cell can be horizontally transferred [4,5]. Consequently more reliable phylogenies can be built based on approaches exploiting larger sequence samples, for example, whole-genome sequence [11,12]. Moreover, homologous recombination might well complicate the inference of the true evolutionary history of the genomes under study [12,13]. Many closely related bacteria exchange a significant amount of DNA sequence via homologous recombination through highly similar patches throughout their genome sequence [14]. Therefore, different regions within those genomes might well have different evolutionary histories that cannot be reliable captured by phylogenies relying on a single tree topology [12].

In the following section, we describe a comparative analysis between eleven *Salmonella*, three *E. coli *and one *Shigella *strain in order to infer the relative time of insertion of putative HGT events in three strains of the *S. enterica *lineage by implementing a whole-genome sequence based alignment to construct the phylogenetic tree topology of the organisms under study. The relative time of insertion is inferred taking into account the most parsimonious sequence of events, that is, allowing for deletions or independent acquisitions in some of the descendant or ancestral branches. Moreover, we discuss and analyze data suggesting that prophages in the *Salmonella *lineage are shared only between very recently diverged lineages but that their sequence composition is very similar to their host's. Finally, we describe the implementation of G+C content, the Codon Adaptation Index (CAI) [15] and high order compositional vectors [16] in order to monitor the amelioration process over time.

## Results

### Time distribution of PHA genes

**Figure 1:**
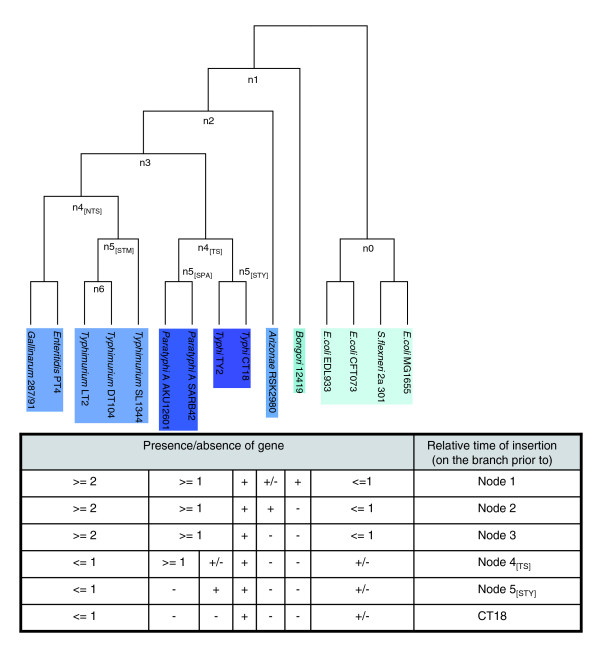
Inferred relative time of insertion of putative horizontally acquired genes, using Typhi CT18 as the query genome. The reference tree topology, based on whole-genome sequence alignment, is shown in the upper section of the figure, with the pseudo-code describing the algorithm for inferring the relative time of insertion shown at the bottom. Node 1 predates the Bongori-Arizonae-Enterica lineage. Node 2 predates the Arizonae-Enterica lineage. Node 3 predates the *S. enterica *lineage. Nodes descendant of node 3 are inferred relative to the query genome: node 4_[TS] _(Typhoidal *Salmonella*) predates the Typhi-Paratyphi A lineage, and node 4_[NTS] _(non-Typhoidal *Salmonella*) predates the Typhimurium-Enteritidis-Gallinarum lineage. Node 5_[STY] _(STY: *S. typhi*) predates the CT18-TY2 lineage, node 5_[SPA] _(SPA: *S. paratyphi *A) predates the SARB42 - AKU_12601 lineage, and node 5_[STM] _(STM: *S. typhimurium*) predates the SL1344-DT104-LT2 lineage. (Note on node 6, relative to LT2: for a fully resolved tree, that is, with fully bifurcating topologies, polytomies, for example, trichotomies, are not allowed. Although Typhimurium LT2 and DT104 are assigned in the same node (node 6), with SL1344 in an apparently separate branch under node 5_[STM]_, the three Typhimurium strains are very close phylogenetically; moreover, there are no genes restricted on branch 6, that is, genes shared between LT2 and DT104 that are absent from SL1344. For these reasons, throughout this analysis, node 6, relative to LT2, is ignored and node 5_[STM] _is considered to be the Typhimurium lineage-specific node.)

**Figure 2:**
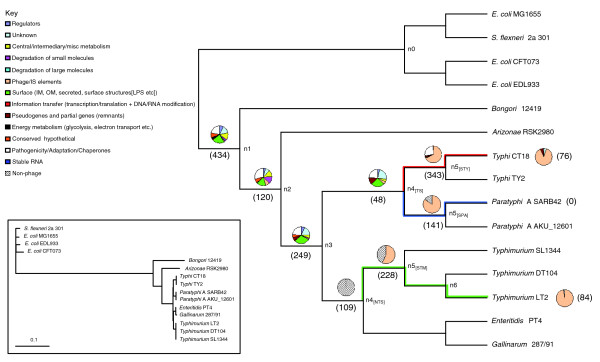
Numerical and functional distribution of PHA genes. The cladogram (main) shows the phylogenetic relationship between the 15 genomes used in this study, ignoring branch length. The topology of the tree is based on whole-genome sequence alignment. The true phylogenetic distance with the respective branch lengths drawn to scale are shown in the phylogram detailed in the inset; the phylogram was built using the *Kimura *2-parameter model. Numbers within parentheses (main) reflect the number of PHA genes. Pie charts on each branch represent the functional classification of genes based on the color-scale detailed in the key. The non-phage functional class (black and white downward diagonal color pattern) was introduced to classify CDSs without color-coded functional classification in their annotation; those CDSs assigned into the 'non-phage' pseudo-class represent CDSs that belong to any of the 13 functional classes apart from the phage class. Numbers of genes on branches 1, 2 and 3 reflect the intersection of the respective number of genes determined on each branch using one of the three query genomes; the same applies for genes assigned to branch 4_[TS]_.

In order to construct the tree topology that best describes the phylogenetic history of the strains studied in this analysis, we implemented the neighbor joining (NJ) [17] and the maximum likelihood (ML) [18] methods. Interestingly, all four substitution models for the NJ method (with and without the *γ*-correction) and the ML method resulted in identical tree topology (Figures 1 and 2). These data suggest that, using whole-genome sequence information, the true phylogeny of the organisms at hand can be captured reliably (see Discussion for more details).

For each of the three query genomes we inferred the total number of PHA genes, as well as their relative time of insertion (Additional data files 1-3). The results are summarized in Table 1 and Figure 2. Using each of the three query genomes, on the branches prior to nodes 1, 2 and 3, we inferred similar numbers of PHA genes for the corresponding relative time of insertion (for the sake of simplicity, from this point on we will refer to the branch prior to node X as branch X). The different number of PHA genes is principally due to small differences in the number of genes in each genome (insertions, deletions, gene remnants) as well as differences in the genome annotation. From this point on we assign on branches 1, 2 and 3 the intersection of the respective number of genes determined on each branch using each one of the three query genomes. Overall, this reciprocal FASTA analysis suggests that approximately 2,500 orthologous genes form a core gene dataset shared by all the 11 *Salmonella *strains; this number reduces to approximately 2,000 orthologous genes shared by the *E. coli*, *S. flexneri *and *Salmonella *strains used in this study (Additional data file 4). Interestingly, this figure is very close to the 2,049 native genes in the *γ*-Proteobacteria proposed by Daubin and Ochman [9].

**Table 1:** A list of PHA genes and their inferred relative time of insertion

*S. typhi *CT18		*S. paratyphi *A SARB42		*S. typhimurium *LT2	
		
Relative time of insertion	PHA genes	Relative time of insertion	PHA genes	Relative time of insertion	PHA genes
Branch 1	493	Branch 1	434	Branch 1	473
Branch 2	124	Branch 2	120	Branch 2	128
Branch 3	316	Branch 3	268	Branch 3	249
Branch 4_[TS]_	62	Branch 4_[TS]_	48	Branch 4_[NTS]_	109
Branch 5_[STY]_	343	Branch 5_[SPA]_	141	Branch 5_[STM]_	228
Branch CT18	76	Branch SARB42	0	Branch LT2	84
Total	1,414	Total	1,011	Total	1,271

This analysis revealed a surprisingly high number of 434 PHA genes inserted at the base of the *Salmonella *lineage (branch 1). Based on two independent previous studies [1,2] the divergence of the *E. coli *and *Salmonella *lineage occurred approximately 100-140 Myr ago. Consequently, putative HGT events on branch 1 represent ancient insertions, close to the divergence of these two lineages and include 76 coding sequences (CDSs) of 'ancient' SPIs, such as SPI-5, SPI-4, a part of SPI-2 (ttr-region), SPI-9, SPI-1 and a part of SPI-3 (magnesium transport ATPase - mgt region).

The *cob *operon of *S. enterica*, which encodes vitamin B12 biosynthesis, has been previously shown to be horizontally acquired in the *Salmonella *lineage following its divergence from the *E. coli *lineage [19,20]. In a later study, Lawrence and Ochman [3] showed, using a model of reverse amelioration, that the *cob *operon was probably introduced into the *Salmonella *lineage 71 Myr ago. The current analysis assigned the *cob *operon to branch 2, which predates the divergence of *S. arizonae *from the *S. enterica *lineage. Based on the data available, we can infer that the divergence of *S. arizonae *from the *S. enterica *lineage occurred approximately 100-71 Myr ago, and further suggest that the 120 inferred PHA genes assigned to branch 2 have an absolute time of insertion of the same order of magnitude.

On branch 3 (*S. enterica *lineage), there are 249 inferred PHA genes. On this branch are found SPIs that are restricted to the *S. enterica *lineage, such as part of SPI-3 (3' end), part of SPI-10 (fimbrial-sef operon), SPI-6, SPI-16 and SPI-17. Finally, on more recent branches, that is, branch 5_[STY] _(STY: *S. typhi*), branch 5_[SPA] _(SPA: *S. paratyphi *A), branch 5_[STM] _(STM: *S. typhimurium*) and strain-specific genes (relative to each of the three query genomes), we have inferred a significant number of putative HGT events, which are mainly dominated by CDSs that belong to annotated prophage structures (discussed in more detail below).

### Functional analysis of PHA genes

Implementing a classification of 14 functional classes (listed in Figure 2), we were able to assign each of the PHA genes, with a given relative time of insertion, into one of the 14 color-coded functional classes. The results are summarized, via pie charts assigned to each branch, in Figure 2. Overall, from this functional classification, it is clear that PHA genes on branches 1-3, branch 4_[TS] _(Typhoidal *Salmonella*) and branch 4_[NTS] _(non-Typhoidal *Salmonella*) show a wide distribution over almost all the 13 functional classes (for example, cell-surface, regulation, central metabolism, pathogenicity), while gene remnants/pseudogenes are mainly restricted to recently diverged lineages, that is, the *S. enterica *species. Moreover, CDSs that belong to annotated structures of prophages (light pink-colored functional class in Figure 2) are predominant in very recent lineages (that is, on branches 5_[STY]_, 5_[SPA]_, 5_[STM]_, or strain-specific CDSs).

On branch 4_[TS]_, which predates the Typhi-Paratyphi A divergence, overall, 24% of genes have unknown functions, 26% encode cell surface-related components, 11% are remnants/pseudogenes and 24% are related to pathogenicity or adaptation (Additional data file 5). Also on this branch are the CDSs of a previously uncharacterized 8.5 kb genomic island (GI; position 2,187,521-2,195,992 bp; Additional data file 6) of very low G+C (36.29%) content that encodes 16 CDSs (STY2349-STY2364 in CT18) of unknown function without significant similarity to previously annotated CDSs. Furthermore, this novel GI does not have any of the 'classic' GI-related features, for example, direct/inverted repeats, an integrase gene or insertion adjacent to an RNA locus. Details about the composition of this putative GI and other genes assigned to branch 4_[TS] _will be discussed in the following section.

The functional analysis of the PHA genes assigned to recent branches (branches 5_[STY]_, 5_[SPA]_, 5_[STM] _and strain-specific) is in line with a previous study focused on *E. coli *MG1655 showing that Insertion Sequence (IS) elements and prophage remnants represent mostly very recent insertion events in MG1655 [21]; the same study suggests that very few acquired DNA sequences are maintained for more than 10 Myr in the genome of *E. coli *MG1655. In the current study, no complete, intact prophage structures inserted at the base of the *Salmonella *lineage are present in all 11 *Salmonella *strains, nor are there any prophages inserted in the *S. enterica *lineage that are shared between the Typhi, Paratyphi A and the Typhimurium strains. Using Typhi CT18 as a query genome, on branch 5_[STY]_, 67% (231) of CDSs belong to prophage structures, while 93% (71) CT18-restricted CDSs are of phage origin. Similarly, in the case of Typhimurium LT2, 57% and 98% of genes that are on branch 5_[STM] _and LT2-restricted, respectively, belong to annotated prophage structure. In the lineage of Paratyphi A, 85% of CDSs acquired on branch 5_[SPA] _are of phage origin; interestingly, there are no SARB42-specific CDSs relative to Paratyphi A AKU_12601.

In a previous study, Thomson *et al*. [22] provided data showing that many prophage structures present in Typhi CT18 are predicted to be Typhi-specific, further suggesting that these bacteriophages have a level of specialization for their host and play a key role in generating genetic diversity in the *S. enterica *lineage. Moreover, the same authors suggested that Typhi has indeed a unique pool of prophage elements that distinguish it from other serovars, in contrast with the *Salmonella *specific SPIs, which show a wider distribution within the *Salmonella *lineage [23].

Generally, in microbial genomes, some PHA genes are retained over long evolutionary distances and, therefore, contribute to species diversification [24,25], while PHA genes that might be detrimental, or not advantageous, for the host are rapidly removed [21,26]. Horizontally acquired DNA is more likely to be deleted than are native, core genes; for example, prophage structures often harbor direct repeats forming their endpoints (that is, attL and attR) that can, via homologous recombination, be used to efficiently remove those 'parasitic' elements. Furthermore, some prophage genes can be detrimental (for example, the *N *gene of bacteriophage *λ*), neutral (for example, integrases) or advantageous (for example, immunity repressors) [26]. Based on this model, parasitic-detrimental DNA sequences (for example, prophage elements) are removed before killing the cell [26]. This bias of deletion over insertion [27] can equilibrate HGT events, and this is further supported by the comparable genome size of closely related genomes [28]. Overall, the current study suggests that, indeed, prophage structures cannot be retained for a long time in the *Salmonella *lineage, while complete, intact prophage structures represent very recent insertions in the Typhi, Paratyphi A and Typhimurium lineages, which, based on their impact (detrimental, neutral or advantageous) on the host, will eventually be retained or removed from those genomes.

### Compositional analysis of PHA genes

The aim of the compositional analysis in this study was to determine if there is any clear trend for genes assigned to relatively old branches in the reference tree topology to show sequence composition closer (compared to more recent insertions) to the average composition of the host genome, thus supporting the effect of amelioration as a time-dependent process. It should be noted that because this analysis is focused on the effects of the amelioration in the *Salmonella *lineage, which diverged fairly recently from *E. coli *and the rest of the enteric bacteria, we expect to identify, if any, mild effects of the amelioration on the sequence composition of the gene datasets under study. For example, Daubin and Ochman [9], applying a similar approach on a much broader phylogenetic sample (the *γ*-Proteobacteria), showed a strong correlation between the G+C content and different phylogenetic depths in their reference tree topology.

**Figure 3:**
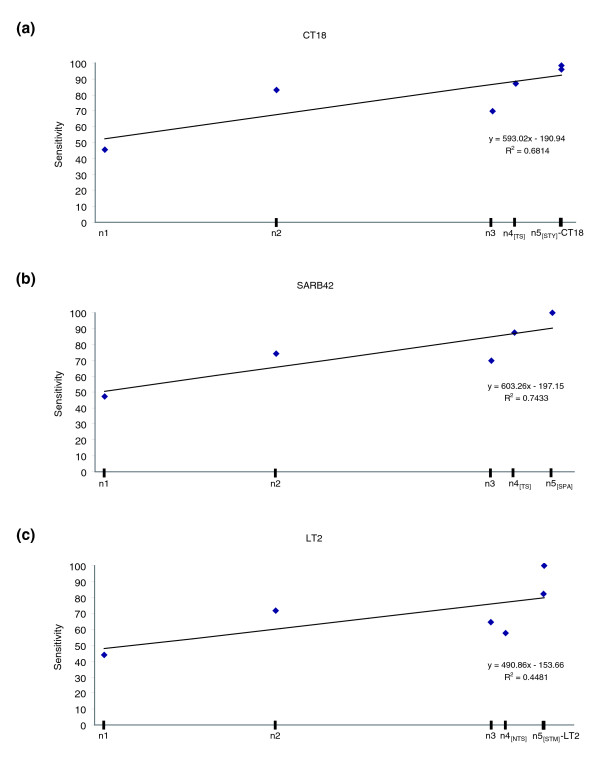
Sensitivity versus relative time of insertion. Sensitivity of the alien_hunter algorithm, which implements the IVOMs method, versus the inferred relative time of insertion for the three query genomes: **(a) ***S. typhi *CT18, **(b) ***S. paratyphi *A SARB42, **(c) ***S. typhimurium *LT2. The nodes on the X-axis are scaled according to the respective branch lengths of the tree topology shown in the inset of Figure 2. Regression analysis is provided within the three graphs.

**Figure 4:**
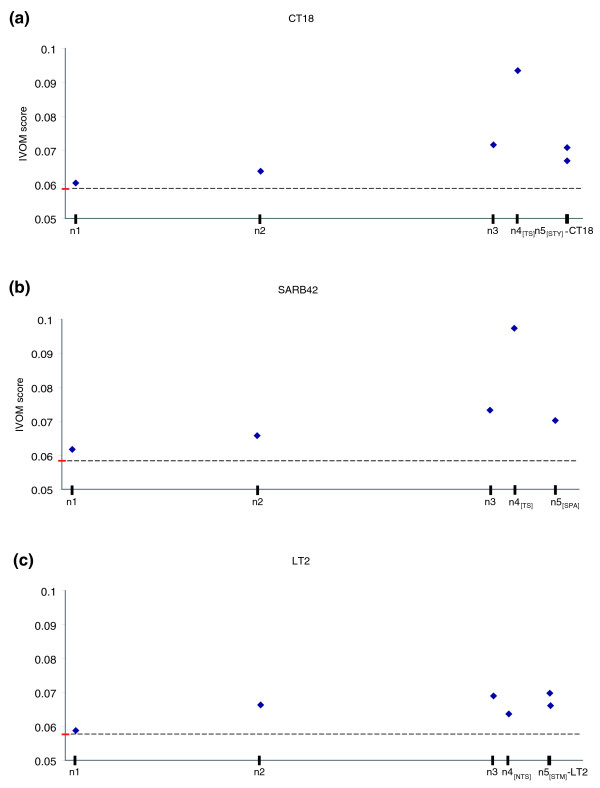
IVOMs score versus relative time of insertion. Average score, taking into account higher order compositional biases, of putative horizontally acquired genes versus the inferred relative time of insertion in the three query genomes: **(a) ***S. typhi *CT18, **(b) ***S. paratyphi *A SARB42, **(c) ***S. typhimurium *LT2. The score is calculated implementing the IVOMs method. The average score for the three query genomes is highlighted in red (the dashed line is provided for ease of comparison). The nodes on the X-axis are scaled according to the respective branch lengths of the tree topology shown in the inset of Figure 2.

As a starting point for the compositional analysis of PHA genes, we applied the *alien_hunter *algorithm, which implements the interpolated variable order motifs (IVOMs) method [16], to the three query genomes, and performed a benchmarking analysis of its sensitivity versus the inferred relative time of insertion of PHA genes; the results are summarized in Figure 3. Overall, it can be concluded that the sensitivity of this HGT prediction method correlates strongly with the relative time of insertion. Indeed, in all the three query genomes, regression analysis showed a correlation (0.45 ≤ R^2 ^≤ 0.75) between the sensitivity and the relative time of insertion. For example, PHA genes inserted at the base of the *Salmonella *lineage (for example, on branch 1) can be identified with a false negative (FN) rate of 0.55 while more recent insertions have a much lower FN rate of 0-0.2. It is worth noting that the high sensitivity of *alien_hunter *on very recent branches is in contrast with the drop in the IVOMs score distribution (Figure 4); the majority of the PHA genes assigned to these branches belong to prophage structures and, consequently, their clustering and not their composition should mainly explain the high sensitivity of this algorithm on these branches. It is important to mention that the analysis of the sensitivity of this algorithm relies on the assumption that all the PHA genes identified in the current analysis are true horizontally acquired genes and the conclusions drawn about its performance are specific for this set of PHA genes.

**Figure 5:**
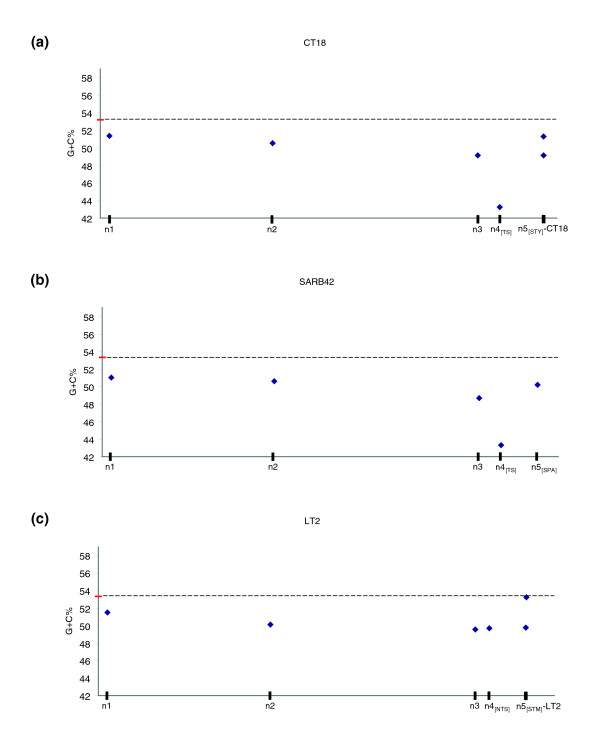
G+C content versus relative time of insertion. Average G+C content of putative horizontally acquired genes versus the inferred relative time of insertion in the three query genomes: **(a) ***S. typhi *CT18, **(b) ***S. paratyphi *A SARB42, **(c) ***S. typhimurium *LT2. The average G+C content for the three query genomes is highlighted in red (the dashed line is provided for ease of comparison). Error bars could not be visualized (the standard deviation is in the range 0.05-0.08). The nodes on the X-axis are scaled according to the respective branch lengths of the tree topology shown in the inset of Figure 2.

Using the G+C content, both overall and codon position specific, as well as higher order compositional biases implementing the IVOMs method, we were able to monitor the amelioration process versus the relative time of insertion of PHA genes (Figures 4 and 5). Using Typhi CT18 and Paratyphi A SARB42 as query genomes, this analysis revealed that there is a clear correlation (R^2 ^= 0.98 for branches 1-3, R^2 ^= 0.65 for branches 1-4_[TS]_) between the G+C content or the IVOMs score of PHA genes and the relative time of their insertion on the earlier branches; however, this strong correlation seems to 'break down' in the case of very recent putative HGT events, that is, insertions that took place after the divergence of Typhi and Paratyphi A lineages (Figures 4 and 5). For example, genes assigned to branches 1 and 2 show average G+C content of 51.4% and 50.6%, respectively, close to the average gene G+C content of 53.2% and 53.3% (for CT18 and SARB42, respectively). The same observation becomes much clearer when calculating higher order compositional biases (Figure 4). Based on the IVOMs score, genes on branches 1 and 2 have average scores of 0.06 and 0.063, respectively, while more recently acquired genes, that is, on branches 3 and 4_[TS]_, have scores of 0.072 and 0.093, respectively; the average, genome-wide IVOMs score in Typhi CT18 is 0.059.

A similar observation can be made for Typhimurium LT2. More specifically, there is a very strong correlation (R^2 ^= 0.89) between G+C content or IVOMs score and the relative time of insertion, which breaks-down on branches descendent of node 3 (Figure 4 and 5). More specifically, the average G+C content of genes assigned to branches 1, 2 and 3, is 51.5%, 50% and 49.6%, respectively, while for genes on branch 4_[NTS]_, the average G+C content is 49.7%. Similarly, using the IVOMs method, the corresponding scores for the four branches are 0.059, 0.066, 0.069 and 0.064, respectively.

**Figure 6:**
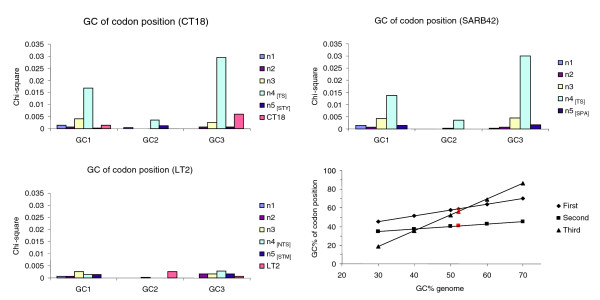
G+C content over the three codon positions. Chi-square values of G+C content over the three codon positions for genes assigned to lineages of increasing depth in the reference tree topology. Chi-square values were calculated using the expected G+C codon-position values derived from the three linear equations provided by Lawrence and Ochman [3] (see equations 13, 14 and 15 therein). At the right-bottom side of the figure, the correlation between genomic G+C content and G+C content at the three codon positions based on the data provided by Muto and Osawa [50] is provided. Genes that are still under the amelioration process are expected to deviate from those expected values. The expected G+C content for each codon position in the *Salmonella *lineage is highlighted in red.

PHA genes assigned to branch 4_[TS] _on the Typhi-Paratyphi A lineage show a very strong compositional deviation, indicated both by their very low G+C content of 43.3% (gene average: 53.2%) and the IVOMs score of 0.093 (genome average: 0.059). Furthermore, the codon-position specific G+C content of genes assigned to branch 4_[TS] _deviate strongly (GC_1 _= 49%, GC_2 _= 37%, GC_3 _= 43%; Figure 6) from the expected values (GC_1 _= 59%, GC_2 _= 41%, GC_3 _= 56%, respectively) based on the three linear equations provided by Lawrence and Ochman [3] (see equations 13, 14 and 15 therein). The G+C content of the second codon position is generally very constrained to similar values across species [3], given that most possible nucleotide substitutions would result in a change in the encoded amino acid residue (non-synonymous substitutions). Interestingly, genes assigned to branch 4_[TS] _in the Typhi-Paratyphi A lineage also show a significant deviation in this compositionally well-conserved codon position, possibly suggesting a distantly related donor genome.

Codon usage analysis revealed that genes on branch 4_[TS] _show a bias towards A+T rich codons (Additional data file 7). For example, the 'AAA' codon is overrepresented in CDSs of this branch, compared to its average frequency in the genome; the AAA codon (encoding lysine) has been previously shown to be overrepresented in highly expressed genes [15]. To test further whether genes on this branch deviate compositionally due to their highly expressed pattern, rather than their alien origin, we performed a CAI analysis (summarized in Table 2). It can be clearly seen that genes on branch 4_[TS] _deviate compositionally from the genome background composition, more likely due to their alien origin, rather than their high rate of expression, representing the 'left ear' in the 'rabbit-like' codon bias versus CAI plot described in [29]. Indeed, genes on branch 4_[TS] _show an average CAI value of 0.221, significantly lower (*p *value = 4.95 10^-13^) than the average gene CAI value (= 0.31) and much lower than the CAI values of highly expressed genes, for example, the genes encoding for ribosomal proteins (CT18, 0.554; SARB42, 0.560; LT2, 0.561) and aminoacyl-tRNA synthetases (CT18, 0.437; SARB42, 0.453; LT2, 0.434). Furthermore, the CAI analysis revealed that genes inferred in this study to be PHA do not show CAI values of highly expressed genes and, overall, their CAI values are significantly lower (*p *value = 3.75 10^-74^) than the average gene CAI values.

**Table 2:** Average CAI values for genes of different inferred relative time of insertion for the three query genomes

*S. typhi *CT18		*S. paratyphi *A SARB42		*S. typhimurium *LT2	
		
Genes	CAI	Genes	CAI	Genes	CAI
PHA on branch 1	0.264	PHA on branch 1	0.264	PHA on branch 1	0.264
PHA on branch 2	0.258	PHA on branch 2	0.258	PHA on branch 2	0.258
PHA on branch 3	0.256	PHA on branch 3	0.256	PHA on branch 3	0.256
PHA on branch 4_[TS]_	0.221	PHA on branch 4_[TS]_	0.221	PHA on branch 4_[NTS]_	0.275
PHA on branch 5_[STY]_	0.283	PHA on branch 5_[SPA]_	0.297	PHA on branch 5_[STM]_	0.269
PHA on branch CT18	0.282	PHA on branch SARB42	NA	PHA on branch LT2	0.307
All genes	0.310	All genes	0.315	All genes	0.313
Ribosomal	0.554	Ribosomal	0.560	Ribosomal	0.561
tRNA synthetase	0.437	tRNA synthetase	0.453	tRNA synthetase	0.434

Average CAI values for all genes in the genome, ribosomal protein coding and aminoacyl-tRNA synthetase genes, are also provided as a reference. Genes ≤300 bp were excluded. The reference gene set of highly expressed genes was the one proposed by Sharp and Li [49] using the genome of *E. coli*.

Overall, using any of the three query genomes (CT18, SARB42, LT2), this analysis indicates that very recent acquisitions, for example, on branches 5_[STY]_, 5_[SPA]_, 5_[STM]_, seem to have been 'ameliorated' to an equal degree as acquisitions on older branches, for example, branches 1 and 2; moreover, in the case of the LT2 genome, strain specific acquisitions (see the LT2 branch) show sequence composition very close to the genome composition. Very recent acquisitions are expected to deviate strongly from the host backbone composition, unless the donor is very close compositionally to the host. Amelioration, a time-dependent process, can not have significantly affected their sequence composition, which should still reflect mostly the donor rather than the host specific compositional signature. However, recent acquisitions identified in this study either show very close composition to the host backbone composition (for example, PHA genes on the LT2 branch have an average G+C content of 53.26%, very close to the gene average G+C content of 53.33%), or deviate compositionally to an equal degree as PHA genes acquired on older branches (for example, the G+C content of PHA genes in CT18 on branches 1 and 5_[STY] _is 51.4 and 51.3, respectively). Similarly, the G+C content of PHA genes in SARB42 on branches 2 and 5_[SPA] _is 50.6% and 50.2%, respectively.

Interestingly, branches descendant of nodes 4_[TS] _and 4_[NTS] _are dominated by genes of phage origin (57% to 98% of genes at the given relative time of insertion; Figure 2). For example, on branch 5_[STY]_, 67% of Typhi CT18 genes assigned to this branch belong to one of the six prophage structures present both in Typhi CT18 and TY2. On branch 5_[STY]_, the G+C content of SPI-7 and the phage-related gene is 50.87% and 51.98%, respectively. In a previous study, it was shown that the last common ancestor of Typhi existed 15,000-150,000 years ago, during the human hunter-gatherer period [30]; consequently, PHA genes assigned to branch 5_[STY] _have a time of insertion of the same order of magnitude. Similarly, in Typhimurium LT2, there are two prophage (Fels-1, Fels-2) structures that represent very recent acquisitions (LT2-specific), and are absent from the other two Typhimurium strains. CDSs of these prophage elements have an average G+C content of 53.57% and 52.94%, respectively, while their CAI value is 0.307, very close to the LT2 genome average CAI of 0.313.

## Discussion

The aim of this analysis was to study the distribution of PHA genes in a time-dependent manner, that is, to infer the relative time of insertion based on the reference tree topology, throughout the *Salmonella *lineage, applying an extensive comparative analysis between eleven *Salmonella*, three *E. coli *and one *Shigella *strain. The selection of four genome sequences that form an outgroup of the *Salmonella *lineage was made in order to differentiate more reliably gene loss from gene gain, two mechanisms that could explain the presence of a gene in one lineage and its absence from a sister, closely related lineage. However, because the *E. coli *and *Salmonella *lineages represent very closely related, sister lineages, the 434 PHA genes inferred to have been acquired at the base of the *Salmonella *lineage might equally represent deletion events in the *E. coli *lineage subsequent to the common ancestor with *Salmonella*. To investigate further this alternative scenario, we used a set of three more distantly related enteric outgroup genomes: *Erwinia carotovora *SCRI1043 (EMBL: BX950851) [31], *Yersinia enterocolitica *8081 (EMBL: AM286415) [32] and *Y. pseudotuberculosis *IP32953 (EMBL: BX936398) [33]. Less than 5% of the 434 PHA genes inferred to have been acquired on branch 1 have orthologous genes present in this distant outgroup (data not shown). These data suggest that the majority (>95%) of the 434 PHA genes most likely represent true HGT events that occurred quite early in the evolution of the *Salmonella *lineage, rather than deletion events in the *E. coli *lineage.

In the current study we exploited a much larger sequence sample, that is, the whole genome sequence, rather than selected gene/protein sequences, to serve as 'molecular chronometers'; thus, the phylogenetic signature seems to be strong enough for the NJ and ML methods to result in identical tree topologies, inferring the same phylogenetic history for the query genomes at hand. However, care should be taken when interpreting whole-genome sequence based phylogenies, since extensive HGT events, homologous recombination or other homoplastic events might well obscure the true phylogenetic histories of the genomes under study [12], whose phylogenies may, therefore, be more efficiently described using phylogenetic nets rather than single tree topologies [11,34,35]. It is worth noting that whole-genome based phylogenetic approaches capture the 'overall' phylogenetic signal based on whole chromosome sequences. In the case of very closely related organisms, for example, strains of the same serovar, minor differences in terms of gene content (for example, prophages, GIs) cannot be reliably represented in the 'overall' phylogenetic signal. In other words, whole genome-based phylogenies focusing on a wide range of strains may suffer from low resolution in the case of very closely related genomes. Moreover, mobile elements may show similarity on the sequence level (for example, prophages) but differ on the structural level (that is, different phage types). Relying on sequence information only, these seemingly similar mobile elements will bias the relatedness of closely related strains (for example, the three Typhimurium strains used in this study).

The reason why we pursued a comparative rather than a compositional based approach (that is, defining PHA genes based simply on their compositional deviation, but ignoring their distribution throughout the lineage of interest) was the fact that compositional based approaches frequently underestimate the true number of HGT events [3], either due to the amelioration process, in the case of ancient insertions, or due to compositionally similar donor genomes, in the case of new insertions. The current comparative analysis suggests that approximately 30%, 25% and 28% of protein-coding sequences in Typhi CT18, Paratyphi A SARB42 and Typhimurium LT2, respectively, represent putative HGT events. The distribution of these PHA genes on different branches of the reference tree topology reveals that approximately 35% to 40% of them were acquired at the base of the *Salmonella *lineage (branch 1), very close to its divergence from *E. coli*, reflecting perhaps the acquisition of genes that enabled the exploration of new niches, for example, the acquisition of SPI-1, which enabled *Salmonella *to invade epithelial cells [36]. Moreover, 20% of those genes were acquired at the base of the *S. enterica *lineage (branch 3); overall, 60% to 70% were inserted after the divergence of the *Salmonella *from the *E. coli *lineage and prior to the divergence of the *S. enterica *subspecies. This suggests that approximately 60% to 70% of the putative HGT events are probably shared between most of the subspecies of the *S. enterica *lineage.

Based on the functional classification of genes assigned to branches 1, 2 and 3 that predate the *S. enterica *lineage, it becomes evident that, generally, genes within almost all functional classes, for example, regulation, energy metabolism, cell surface, and virulence-related, have been horizontally acquired. Moreover, genes on branches 1, 2 and 3 show a significant correlation (Pearson correlation coefficient = 0.7-0.92) in the percentage of the corresponding functional classes. For example, there is a fairly constant percentage of genes encoding cell-surface structures (18% to 28%), genes related to pathogenicity and adaptation (22% to 29%) and regulatory elements (4% to 8%). Furthermore, the percentage of genes with unknown function ranges from 8% to 18%, while fragmented gene remnants (pseudogenes) account for 6% and 11% on branches 3 and 4_[TS]_, respectively, with almost no pseudogenes (<0.1%) on branches 1 and 2. The increased number of genes acquired at the base of the *S. enterica *lineage that have been inactivated suggests that some of these early acquired functions are no longer necessary, and are being lost in these serovars. The increased number of pseudogenes (11%) in the Typhi-Paratyphi A lineage that are absent from the Typhimurium lineage supports a genome degradation process via pseudogene formation, suggested to be due to the recent change in niche of these serovars [37].

The compositional analysis of the inferred PHA genes indicates that there is indeed a strong correlation between the time of insertion and amelioration towards the host-specific genomic signature. In other words, anciently horizontally acquired genes have ameliorated more towards the host composition, compared to more recent acquisitions. However, even HGT events inferred to have inserted at the base of the *Salmonella *lineage still preserve some of their donor genome sequence signature, as indicated by their overall and codon-position specific G+C content, suggesting that these genes are still undergoing the amelioration process. On the other hand, in the case of very recent acquisitions that represent mostly insertion of prophage elements, it seems that their sequence composition is already much closer to the host background composition, presumably not due to the amelioration process, since they have been acquired fairly recently, but rather due to an adaptation to the specific sequence signature of the their host.

If we take into account both the absence of complete, intact prophage structures from old branches (1-3, 4_[TS] _and 4_[NTS]_), and the significant compositional similarity of those prophage-related genes to the host sequence composition, when the effects of the amelioration process are expected to be mild, it would be tempting to speculate that prophage elements in the *Salmonella *lineage have undergone an adaptation to specific serotypes. However, this hypothesis does not explain why anciently inserted prophages, for example, those inserted at the base of *Salmonella *lineage prior to the divergence of *S. bongori *and *S. arizonae *from the *S. enterica*, have not been retained in descendent lineages, for example, the Typhi, Paratyphi A and Typhimurium strains. Perhaps anciently inserted bacteriophages at the base of the *Salmonella *lineage carried genes that were either neutral or detrimental, providing no profound advantage to the host, and over time the host has lost those parasitic elements via a deletion process that has left behind molecular fossils of those elements. This observation is further supported by the absence of pseudogenes on very old branches, that is, branches 1 and 2; perhaps the ongoing time-dependent process of deleting redundant or detrimental DNA sequence has already removed a much higher proportion of pseudogenes on very old branches, compared to recent ones, further suggesting that genome degradation is still a continuous process in the *Salmonella *lineage [26].

## Conclusion

Overall, the current analysis has shown that the impact of amelioration, a time-dependent process, is still detectable even in fairly recent HGT events, for example, that occurred 100-140 Myr ago. Moreover it sheds more light on the relative time of insertion of HGT events in the *Salmonella *lineage, and presents data that show that prophage structures are not retained for long periods in the *Salmonella *lineage.

Whether this last observation is related to an ongoing genome degradation process that over time removes redundant or detrimental DNA sequences, equilibrating the horizontal influx of genes and maintaining a fairly constant genome sequence size, still remains to be clarified. Perhaps the study of the very recently acquired prophage elements that seem to account for the majority of the strain or serovar specific genes [22,37], and their impact (detrimental, neutral, advantageous) on the evolution, life-style and host adaptation of the *Salmonella *strains might shed more light on the underlying principles of the observed genome degradation process.

The prophage elements present in the *Salmonella *lineage show a very close sequence composition to the host-specific background composition, strongly suggesting that those parasitic elements have specialized and adapted to their hosts, playing a key role in driving bacterial evolution [22], or even speciation itself, supporting the notion of 'evolution in quantum leaps' introduced by Groisman and Ochman [38]. Overall, the distribution of PHA genes in the *Salmonella *lineage coincides strongly with the divergence of the major *Salmonella *species, underlining the major impact of horizontal transfer on the evolution of the salmonellae.

## Materials and methods

### Phylogenetic analysis

For the 15 genomes analyzed in this study (Table 3), we implemented a whole-genome sequence based alignment approach. Whole genome sequence alignments were made using the MAUVE algorithm [39]. For the construction of the reference tree topology we implemented modules of the PHYLIP package, version 3.65 [40]. More specifically, we used the DNADIST module, which uses nucleotide sequences to compute a distance matrix, under four different models of nucleotide substitution: those of Jukes and Cantor [41] and Kimura [42], the *F84 *model [18,43], and the model underlying the *LogDet *distance [44]. For the first three models we used also the *γ*-based method for correcting the rate heterogeneity among sites. We also used the NEIGHBOR module, which implements the NJ method of Saitou and Nei [17], and the DNAML module, which implements the ML method for DNA sequences [18], using the *γ*-based method. In order to compare the tree topologies obtained, we used the TREEDIST module, which computes tree distances using either branch lengths or node topologies. For the *γ*-based method, we determined the *α *parameter from the datasets, using the TREE-PUZZLE method [45]. The trees obtained were drawn using the TREEVIEW software [46].

**Table 3:** The list of fifteen strains used in this comparative analysis

Organism	Strain	Source	Reference	Accession number
*Escherichia coli *K-12	MG1655	Wisconsin University	[51]	[EMBL: U00096]
*E. coli *O157:H7	EDL933	Wisconsin University	[52]	[EMBL: AE005174]
*E. coli*	CFT073	Wisconsin University	[53]	[EMBL: AE014075]
*Shigella flexneri *serotype 2a	301	Microbial Genome Center of ChMPH	[54]	[EMBL: AE005674]
*Salmonella bongori*	12419	Sanger Institute	[55]	NA
*S. arizonae*	RSK2980	Washington University, St Louis	[56]	NA
*S. enterica *serovar Typhi	CT18	Sanger Institute	[57]	[EMBL: AL513382]
*S. enterica *serovar Typhi	TY2	Wisconsin University	[58]	[EMBL: AE014613]
*S. enterica *serovar paratyphi A	SARB42	Washington University, St Louis	[37]	[EMBL: CP000026]
*S. enterica *serovar paratyphi A	AKU_12601	Sanger Institute	[59]	NA
*S. enterica *serovar Typhimurium	SL1344	Sanger Institute	[55]	NA
*S. enterica *serovar Typhimurium	LT2	Washington University, St Louis	[60]	[EMBL: AE006468]
*S. enterica *serovar Typhimurium	DT104	Sanger Institute	[55]	NA
*S. enterica *serovar Enteritidis	PT4	Sanger Institute	[55]	NA
*S. enterica *serovar Gallinarum	287/91	Sanger Institute	[55]	NA

### Reciprocal FASTA - manual curation

Three *S. enterica *serovars, Typhi strain CT18, Paratyphi A strain SARB42 and Typhimurium strain LT2, were each used as a query genome to infer pair-wise orthologous genes against each of the other fourteen genomes (Table 3). We took the following approach in order to infer the orthologous genes in each pair of genomes compared: Each CDS (a) from the genome (A) was searched, using FASTA [47], against the CDSs of the other genome (B). If the top hit covered at least 80% of the length of both sequences with at least 30% identity, a reciprocal FASTA search of the top hit sequence (b) was launched against the CDSs of the first genome. If the reciprocal top hit was the same as the original query CDS then (a) and (b) are considered orthologous genes of (A) and (B). In a second step, in order to validate the results, we performed a BLASTN and TBLASTX comparison between the 15 genomes, visualized using ACT [48] to curate ambiguous cases, for example, gene remnants (pseudogenes), IS elements and phage-related CDSs, and to check for a syntenic relationship among the putative orthologs.

### Relative time of insertion of PHA genes

In order to differentiate more reliably gene loss from gene gain (HGT), we used a genomic dataset of three *E. coli *and one *S. flexneri *strain that forms the outgroup lineage in our reference tree topology. For example, a gene that is present in the *Salmonella *lineage and absent from *E. coli *MG1655 might well be either a true HGT in the former or deletion in the latter. However, if, for example, the same gene is also present in *E. coli *EDL933 and *E. coli *CFT073, then we can infer more reliably that this event probably represents a deletion (in *E. coli *MG1655) rather than a true HGT in the *Salmonella *lineage. Conversely, a sequence that is confined to one lineage is more likely to have been horizontally acquired than to have been deleted independently from multiple lineages [21]. A pseudo-code of the algorithm applied in order to infer the relative time of insertion of PHA genes, using Typhi CT18 as a query genome, is described in Figure 1. The same approach was followed using Paratyphi A SARB42 and Typhimurium LT2 as query genomes. The table embedded at the bottom of Figure 1 summarizes the parameters used in this study to differentiate gene loss from gene gains events, assuming a maximum parsimony evolutionary model. For example, in the case of CT18, a gene X that has no orthologue in the four outgroups and the *S. bongori *genome but has orthologs in the other nine genomes is more likely to have been acquired on branch 2 (node 2 assignment in Figure 1). Similarly, a gene X in CT18 that has orthologs only in the four outgroup genomes is more likely to represent an independent HGT event in CT18, rather than the result of multiple deletions in the other ten genomes (CT18 assignment in Figure 1).

### Compositional analysis

In order to monitor the level of amelioration with respect to the inferred relative time of insertion for each gene in each of the three query genomes, we calculated the overall as well as the codon-position specific G+C content. Furthermore, to increase the sensitivity of capturing compositionally deviating genes, for example, genes that do not deviate in terms of G+C content but show higher order compositional bias, we implemented the IVOMs method [16]. To differentiate highly expressed from horizontally acquired genes that deviate compositionally, we also performed a CAI analysis, measuring the adaptation of each gene to the codon usage of a reference set of highly expressed genes, proposed by Sharp and Li [49].

## Additional data files

The following additional data are available with the online version of this paper. Additional data file 1 lists the 1,414 PHA genes identified in the *S. typhi *CT18 genome and their relative time of insertion. Additional file 2 lists the 1,271 PHA genes identified in the *S. typhimurium *LT2 genome and their relative time of insertion. Additional file 3 lists the 1,011 PHA genes identified in the *S. paratyphi *A SARB42 genome and their relative time of insertion. Additional file 4 shows the core gene dataset. The Venn diagram illustrates the orthologous genes shared between all the 11 *Salmonella *strains (bold circle in the middle) and the genomes of *E. coli *MG1655, *E. coli *EDL933, *E. coli *CFT073 and *S. flexneri *2a 301. The number highlighted in bold, represents the total number of orthologues genes (core genes) shared between the 15 genomes used in this study. Additional file 5 provides a summary of the functional classification of genes assigned to branch 4_[TS]_, relative to the Typhi-Paratyphi A lineage, using 14 functional classes. The color code for each functional class is detailed at the bottom left of this file. Additional file 6 shows the novel genomic island. The ACT screenshot is of a tBLASTX comparison between five selected *Salmonella *genomes (from top to bottom): *S. typhi *CT18, *S. paratyphi *A SARB42, *S. typhimurium *LT2, *S. enteritidis *PT4 and *S. gallinarum *287/91. Regions within the five genomes with sequence similarity are joined with red colored bands representing the matching regions. The putative GI that is present in Typhi and Paratyphi A genomes is illustrated as a white box. Above the genome of Typhi CT18, the G+C content graph is plotted, with a 0.5 kb sliding window. Additional file 7 shows the codon usage difference of CDSs assigned on branch 4_[TS] _relative to the average codon usage in Typhi CT18. Positive values in the Y axis indicate overrepresentation (blue-colored bars) of certain codons in CDSs of this branch relative to the average codon usage and vice versa.

## Supplementary Material

Additional data file 1 The 1,414 PHA genes identified in the *S. typhi *CT18 genome and their relative time of insertion. Click here for file

Additional data file 2 The 1,271 PHA genes identified in the *S. typhimurium *LT2 genome and their relative time of insertion. Click here for file

Additional data file 3 The 1,011 PHA genes identified in the *S. paratyphi *A SARB42 genome and their relative time of insertion. Click here for file

Additional data file 4 The Venn diagram illustrates the orthologous genes shared between all the 11 *Salmonella *strains (bold circle in the middle) and the genomes of *E. coli *MG1655, *E. coli *EDL933, *E. coli *CFT073 and *S. flexneri *2a 301. The number highlighted in bold, represents the total number of orthologues genes (core genes) shared between the 15 genomes used in this study. Click here for file

Additional data file 5 The color code for each functional class is detailed at the bottom left of this file. Click here for file

Additional data file 6 The ACT screenshot is of a tBLASTX comparison between five selected *Salmonella *genomes (from top to bottom): *S. typhi *CT18, *S. paratyphi *A SARB42, *S. typhimurium *LT2, *S. enteritidis *PT4 and *S. gallinarum *287/91. Regions within the five genomes with sequence similarity are joined with red colored bands representing the matching regions. The putative GI that is present in Typhi and Paratyphi A genomes is illustrated as a white box. Above the genome of Typhi CT18, the G+C content graph is plotted, with a 0.5 kb sliding window. Click here for file

Additional data file 7 Positive values in the Y axis indicate overrepresentation (blue-colored bars) of certain codons in CDSs of this branch relative to the average codon usage and vice versa. Click here for file
