# Supplementary figures and images for: Genetic flux over time in the Salmonella lineage
Source: Genome Biol. 2007 Jun 4;8(6):R100. doi: 10.1186/gb-2007-8-6-r100 (PMC2394748; doi:10.1186/gb-2007-8-6-r100)

**EDL933**

**MG1655**

**CFT073**

**S. flex 2a 301**

**Salmonella**

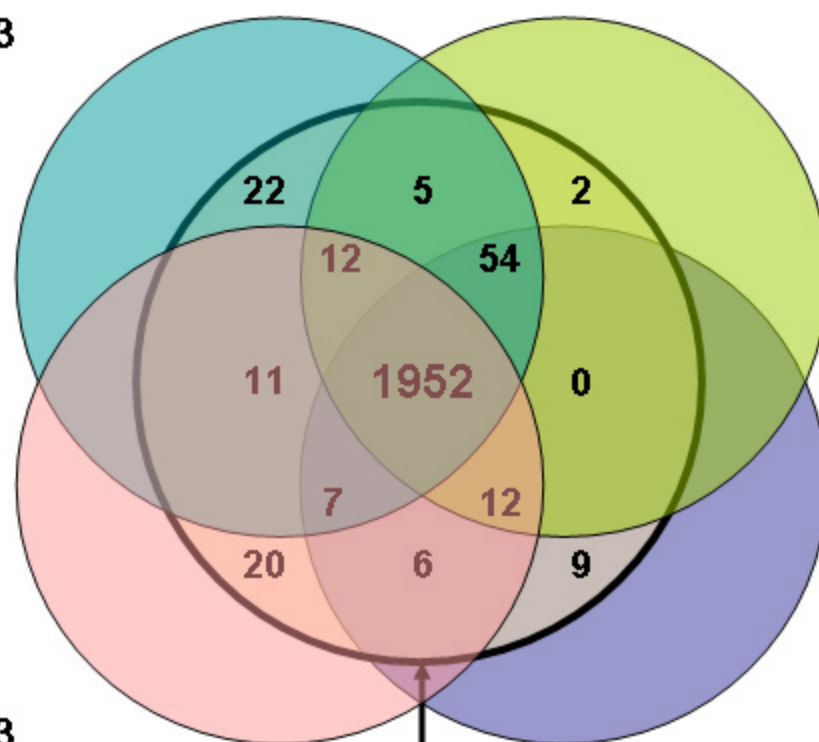

Supplement: Additional data file 4 — The Venn diagram illustrates the orthologous genes shared between all the 11 Salmonella strains (bold circle in the middle) and the genomes of E. coli MG1655, E. coli EDL933, E. coli CFT073 and S. flexneri 2a 301. The number highlighted in bold, represents the total number of orthologues genes (core genes) shared between the 15 genomes used in this study. [file gb-2007-8-6-r100-S4.pdf]

GC Content (%) Window size: 598

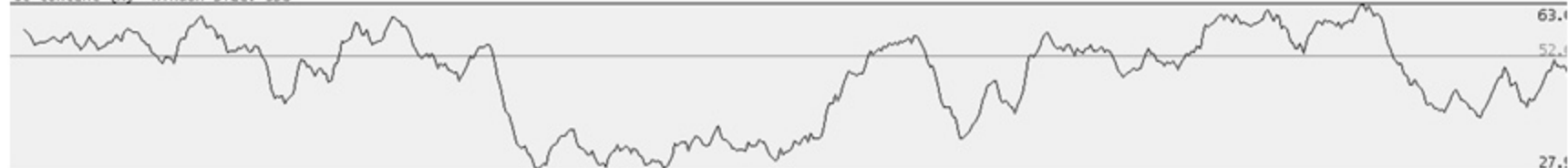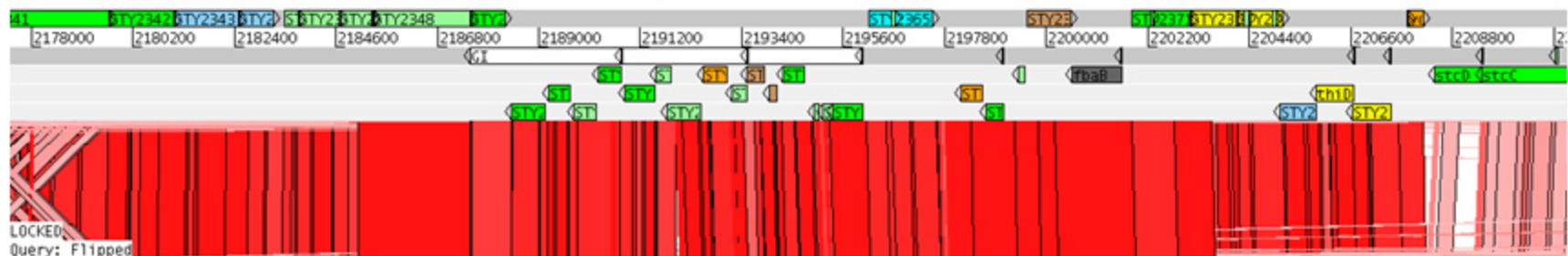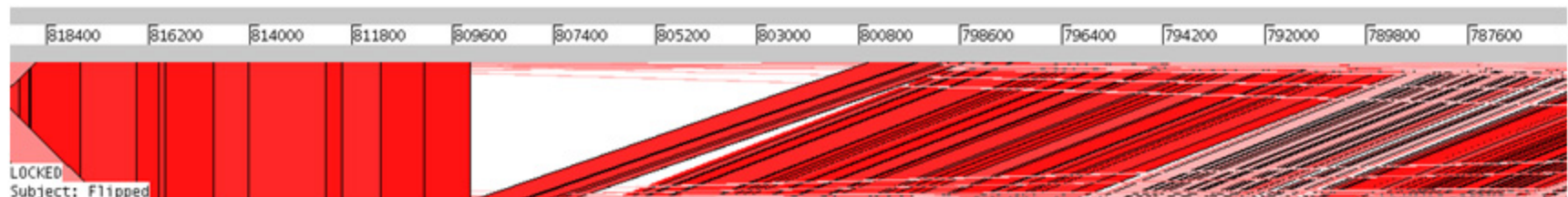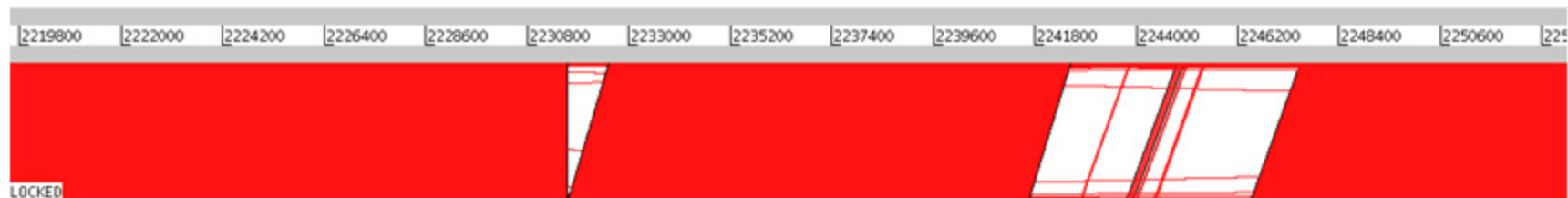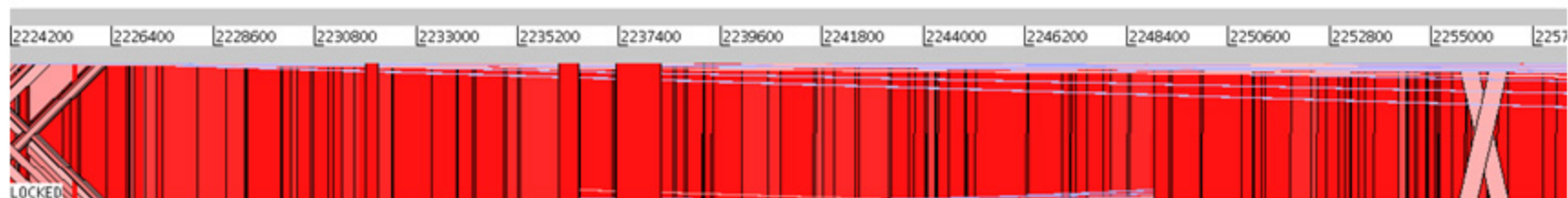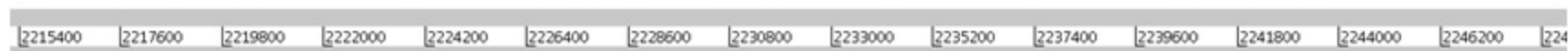

Supplement: Additional data file 6 — The ACT screenshot is of a tBLASTX comparison between five selected Salmonella genomes (from top to bottom): S. typhi CT18, S. paratyphi A SARB42, S. typhimurium LT2, S. enteritidis PT4 and S. gallinarum 287/91. Regions within the five genomes with sequence similarity are joined with red colored bands representing the matching regions. The putative GI that is present in Typhi and Paratyphi A genomes is illustrated as a white box. Above the genome of Typhi CT18, the G+C content graph is plotted, with a 0.5 kb sliding window. [file gb-2007-8-6-r100-S6.pdf]

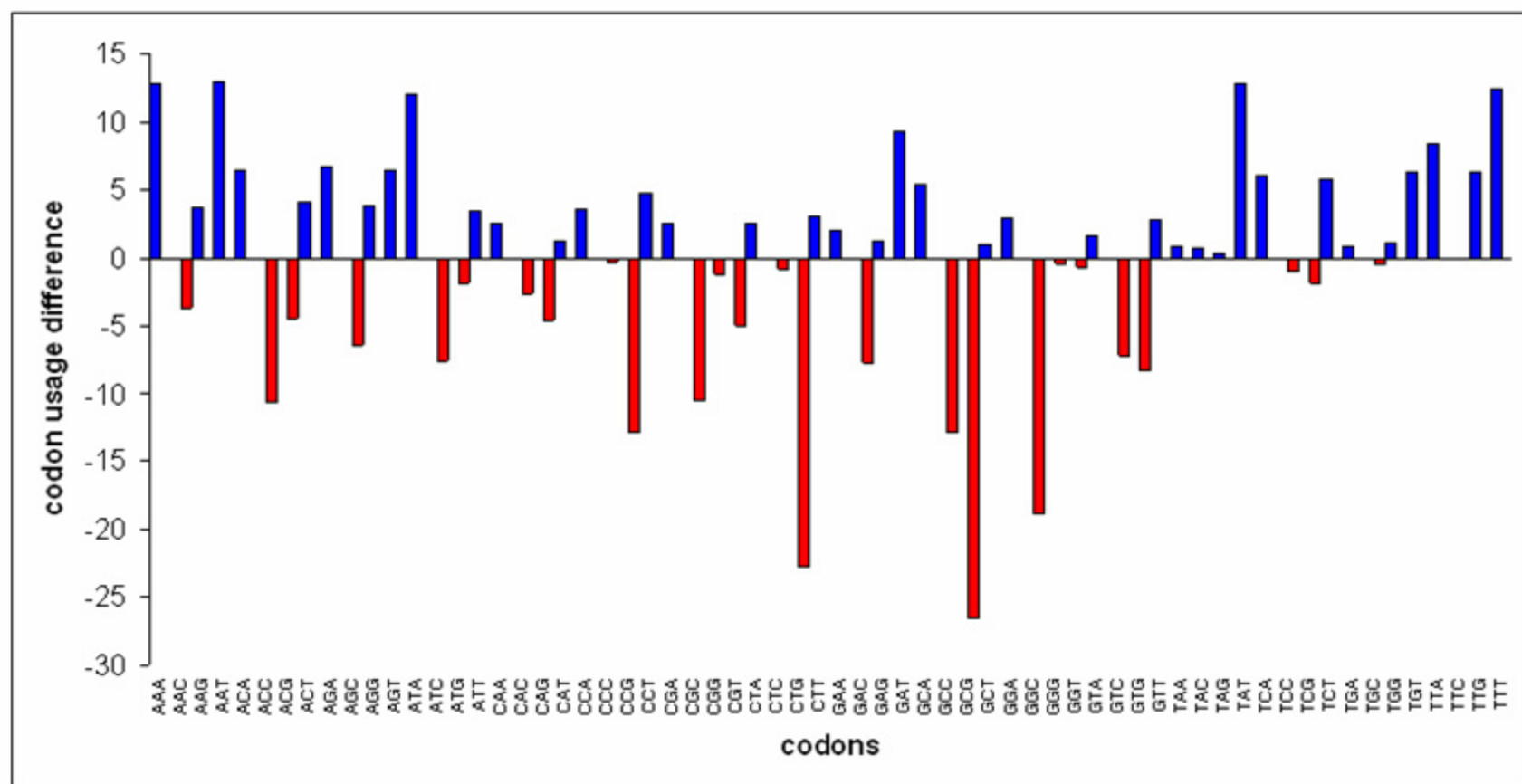

Supplement: Additional data file 7 — Positive values in the Y axis indicate overrepresentation (blue-colored bars) of certain codons in CDSs of this branch relative to the average codon usage and vice versa. [file gb-2007-8-6-r100-S7.pdf]
