# Supplementary material for: Genetic flux over time in the Salmonella lineage
Source: Genome Biol. 2007 Jun 4;8(6):R100. doi: 10.1186/gb-2007-8-6-r100 (PMC2394748; doi:10.1186/gb-2007-8-6-r100)
Supplement: Additional data file 5 — The color code for each functional class is detailed at the bottom left of this file. [file gb-2007-8-6-r100-S5.pdf]

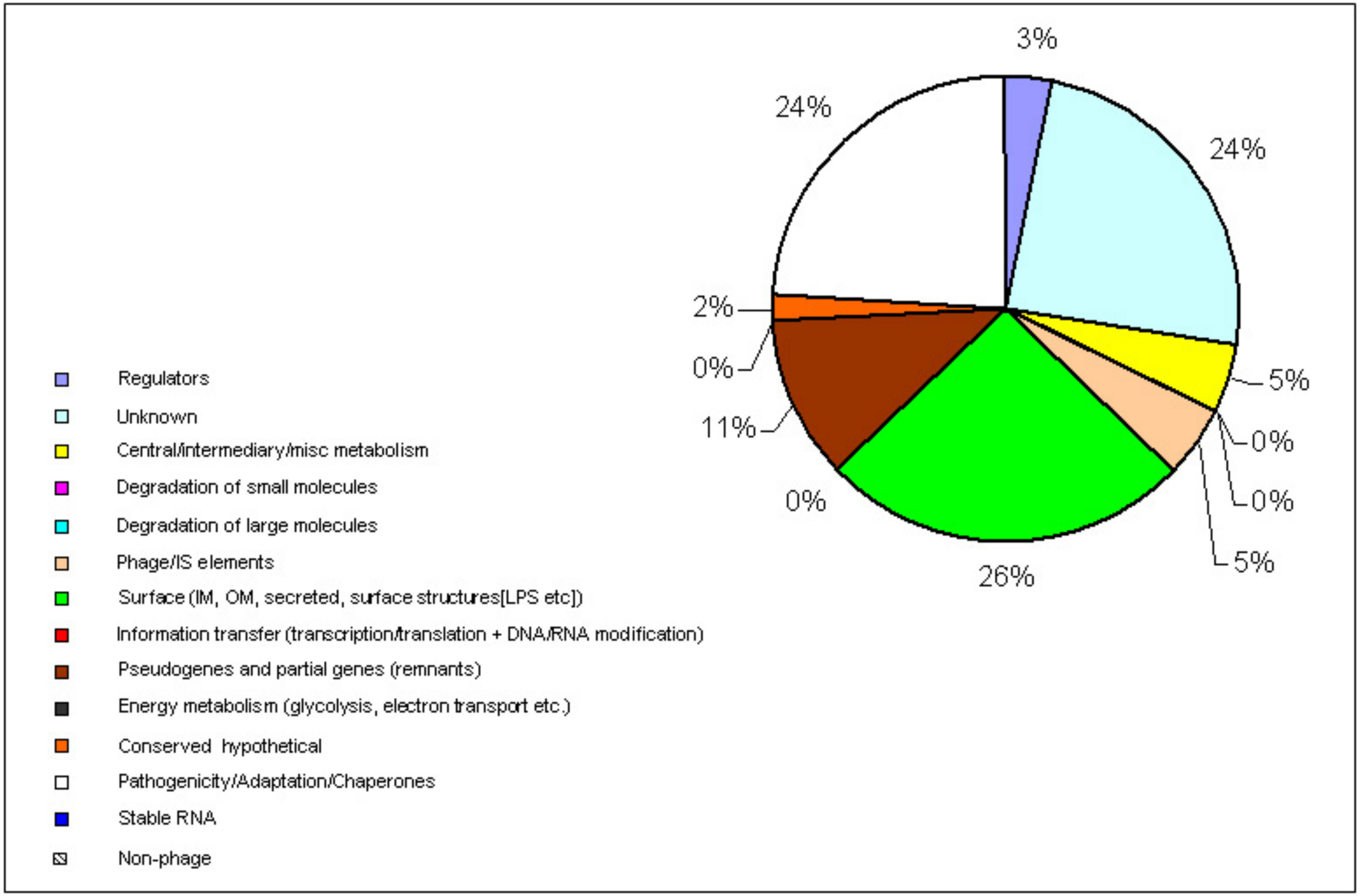
3%
24%
24%
2%
0%
11%
0%
26%
5%
0%
0%
5%
Regulators
Unknown
Central/intermediary/misc metabolism
Degradation of small molecules
Degradation of large molecules
Phage/IS elements
Surface (IM, OM, secreted, surface structures[LPS etc])
Information transfer (transcription/translation + DNA/RNA modification)
Pseudogenes and partial genes (remnants)
Energy metabolism (glycolysis, electron transport etc.)
Conserved hypothetical
Pathogenicity/Adaptation/Chaperones
Stable RNA
Non-phage
